# Supplementary material for: Virulence Traits of Inpatient Campylobacter jejuni Isolates, and a Transcriptomic Approach to Identify Potential Genes Maintaining Intracellular Survival
Source: Microorganisms. 2020 Apr 7;8(4):531. doi: 10.3390/microorganisms8040531 (PMC7232156; doi:10.3390/microorganisms8040531)

Supplementary Table 1. List of primers and annealing temperatures used for PCR analysis.

Target gene	Primers	Sequence	Annealing temp. (ºC)	PCR- product (bp)	Reference
cgtB	*DL39*	5′-tta aga gca aga tat gaa ggt g-3′	54	*420*	*Linton et al. (2000a)*
	cgtBrev	5′-gca cat aga gaa cgc tac aa-3′			
flaB	FlaB3	5′-ata aac acc aac atc ggt gca-3′	53	1670	Smith et al. (1999)
	FlaB4	5′-gtt acg ttg act cat agc ata-3′			
flhB	flhB-q-F	5′-cag gtg cgg atg tgg tga tc-3′	59	101	Müller et al (2006)
	flhB-q-R	5′-cac tcc ttt ggc aac aac cct-3′			
flgB	flgB-q-F	5′-gca cga ttt acc aaa gct gtt tca a-3′	59	123	Müller et al (2006)
	flgB-q-R	5′-cac tgg tgc ttt agc ggg tag a-3′			
flgE2	flE2-q-F	5′-cat ctc acc acg acc tcc tgt tc-3′	55	132	Müller et al (2006)
	flgE2-q-R	5′-gca aaa atc gca atg gct tca-3′			
wlaN	Cj1139cF	5′-tgc tgg gta tac aaa ggt tgt g-3′	55	330	Wassenaar et al. (2002)
	Cj1139cR	5′-aat ttt gga tat ggg tgg gg-3′			
ciaB	CiaB-F	5′-ttt cca aat tta gat gat gc-3′	48	1165	Rivera-Amill et al.(1999)
	CiaB-R	5′-gtt ctt taa att ttt cat aat gc-3′			
iamA	Cia3f	5′-gca caa aat ata tca tta caa-3′	47	518	Müller et al (2006)
	Cia5r	5′-ttc acg act act atg agg-3′			
virB11	VirB11F	5′-gaa cag gaa gtg gaa aaa cta gc-3′	54	709	Bacon et al. (2002)
	VirB11R	5′-ttc cgc att ggg cta tat g-3′			
cadF	cadF-F2B	5′-ttg aag gta att tag ata tg-3′	46	400	Konkel et al. (1999)
	cafF-R1B	5′-cta ata cct aaa gtt gaa ac-3′			
docA	docA1	5′-ata agg tgc ggt ttt ggc-3′	48	725	Müller et al (2006)
	docA2	5′-gtc ttt gca gta gat atg-3′			
docB	docB1	5′-cgg aga gtt tag agg cac c-3′	53	1418	Müller et al (2006)
	docB2	5′-ccg caa att cca tag cag-3′			
docC	docC1	5′-tga gct acg cta tca ttg-3′	51	1835	Müller et al (2006)
	docC2	5′-gct tac gct atg ggt tgg-3′			
cdtB	WMI-R	5′-gtt ggc act tgg aat ttg caa ggc-3′	58	495	Bang et al. (2003)
	Vat2	5′-gtt aaa atc ccc tgc tat caa cca-3′			
cstII	cstII-IIIF	5’-gta ttt aga tgy art car ttt tat ttt gaa g-3’	55	624	This study
	csIIR	5’-tat grt aaa att tga att taa att tgg ygc-3’			
csIII	cstII-IIIF	5’-gta ttt aga tgy art car ttt tat ttt gaa g-3’	51	793	This study
	cstIIIR	5’-tat ttt tat ttg cat att ttt cct tta agt ag-3’			
hcp	hcp_fw	5’-caagcggtgcatctactgaa-3’	60	463	Harrison et al.(2014)
	hcp_rev	5’taagctttgccctctctcca-3’			
cj1373	cj1373_fw	5’-ctt aaa gcc tga tac agt tt-3’	51	140	This study
	cj1373_rev	5’-gta tct aga ctt tgt ttc gt-3’			
cj0182	cj0182_fw	5’-gct att gct att cct tat gt-3’	51	201	This study
	cj0182_rev	5’-act aag tcc taa act ctc ta-3’			
cj1668	cj1668c_fw	5’-gct tta aat gtg tca gca tt-3’	51	179	This study
	cj1668_rev	5’-gga tca act ttt gca aca at-3’			
cj1637c	cj1637c_fw	5’-cct tta gta ggg ctt ata at-3’	51	234	This study
	cj1637c_rev	5’-agt tgc ttt ttc aaa ttc cc-3’			
cj1533c	cj1533c_fw	5’-ggc cta aga aat aat ctt tg-3’	51	194	This study
	cj1533_rev	5’-cgt agc ttg atc aaa tca at-3’			
cj0428	cj0428_fw	5’-cgc aaa act caa att caa att taa g-3’	54	188	This study
	cj0428_rev	5’-gcg ttc aat aaa tca ttt aac aaa g-3’			

Supplementary Table 2. Transcriptional analysis of *C. jejuni* CjTD-119 ORFs during invasion.

Only genes with at least 1.5-fold up- and downregulation detected after normalization were considered for further analysis with the exception of certain (*) genes. In this table, generally the upregulated genes are considered, but in some cases the unchanged or downregulated genes are also demonstrated to get a whole picture of a certain mechanisms. The color code represents the degree of the up-or downregulation. The significantly upregulated and downregulated genes are signed with green and red color, respectively. The orange color represents the not significantly changed genes. CjTD-119 hour 3, normal culture bacterial—trimmed RNA-Seq—Normalized expression values (a); CjTD-119 hour 3, normal culture bacterial—trimmed RNA-Seq—Unique gene reads (b); CjTD-119 hour 3, during invasion bacterial—trimmed RNA-Seq—Normalized expression values (c); CjTD-119 hour 3 during invasion bacterial—trimmed RNA-Seq—Unique gene reads (d). Feature IDs are presented in the last column. They were both compared to the NCTC genes and the genes of CjTD-119. Only genes with at least 1.5-fold up- and downregulation detected after normalization were considered for further analysis. Exceptions from that (genes marked with *) were also chosen in order to obtain a whole picture of the changes concretely regarding to the virulence associated and flagellar genes. ORFs are organized in sections containing the following functional categories: A. Membranes / surface molecules / lipoproteins / porins / transport / binding; B. Adherence / Colonization; C. Bacterial shape determinant genes; D. Capsule; E. LOS / Invasion; F. Iron acquisition; G. Hypothetical proteins; H. Invasion; I. Colonization; J. Regulator / Signal transduction; K. Toxin; L. Chemotaxis; M. Chaperones / stress responses; N. Protein synthesis / modification / secretion, O. O-linked glycosylation; P. N-linked glycosylation; Q. Colonization / Respiration; R. Energy metabolism; S. Mucin degradation; T. Periplasmic proteins; U. Flagellar proteins; V. Other protein coding ORFs.

Function of gene	Experiment - Fold Change (normalized values)	a	b	c	d	Feature Ids: CjTD-119 or gene / NCTC11168 ref. [1]
**A. Membranes/surface molecules/lipoproteins/porins/transport/binding**						
integral membrane protein	14,346	0,889	5	12,747	14	AA01_00086 / Cj0201c
arsenical pump membrane protein	12,809	1,506	18	19,292	45	arsB / Cj1187c
TonB transport protein	12,168	1,153	8	14,034	19	Cj0181
protein translocase subunit SecE	10,713	52,648	88	564,014	184	secE / Cj0472
integral membrane protein	10,247	0,958	22	9,821	44	AA01_00748 / Cj1373
TerC family integral membrane protein	8,384	4,936	33	41,381	54	AA01_00101 / Cj0186c
membrane protein insertion efficiency factor	7,97	11,336	36	90,346	56	AA01_01612 / Cj0959c
pseudaminic acid biosynthesis protein PseA	7,685	0,189	2	1,456	3	pseA / Cj1316c
membrane protein	6,785	4,243	37	28,792	49	AA01_01417 / Cj0692c /
NLPA family lipoprotein	6,382	7,78	57	49,651	71	AA01_00586 / Cj1200
protein translocase subunit SecY	5,655	27,05	318	152,974	351	secY / Cj1688c
Lipoprotein signal peptidase LspA	5,328	5,71	25	30,457	26	lspA / Cj0361
membrane protein	5,331	6,658	74	35,493	77	AA01_00631 / Cj1245c
integral membrane protein	5,124	3,56	48	18,24	48	AA01_00350 / Cj0801

sodium:dicarboxylate family transmembrane symporter	4,955	18,881	243	93,551	235	AA01_00905 / Cj0025c
poly(A) polymerase family protein	4,658	5,293	55	24,654	50	cca / Cj0789
integral membrane protein	4,538	12,501	70	56,731	62	AA01_00554 / Cj1168c
MATE family transport protein	4,099	2,453	30	10,055	24	AA01_01483 / Cj0619
transmembrane transport protein	4,029	9,197	103	37,058	81	AA01_00105 / Cj0182
lipoprotein thiredoxin	3,79	15,598	73	59,116	54	AA01_01002 / Cj1665
lipoprotein	3,751	27,727	112	104,009	82	AA01_01620 / Cj0950c
OmpA family membrane protein	3,622	6,547	58	23,713	41	AA01_00307 / Cj0599
outer membrane protein Omp50	3,564	13,935	184	49,666	128	omp50 / Cj1170c
MoeB/ThiF family protein	3,564	3,753	23	13,376	16	AA01_00167 / Cj0294
phosphate ABC transporter ATP- binding protein PstB	3,522	2,325	16	8,191	11	pstB / Cj0616
membrane protein	3,328	21,289	137	70,86	89	AA01_01553 / Cj0552
protein translocase subunit SecD	3,209	24,794	364	79,57	228	secD / Cj1093c
OstA family protein	3,137	11,422	49	35,828	30	ostA / Cj0649
protein translocase subunit SecF	3,067	15,178	137	46,547	82	secF / Cj1092c
ExbD/TolR family transport protein ExbD3	2,882	8,836	32	25,466	18	exbD3 / Cj0110
transmembrane protein	2,881	23,041	233	66,372	131	AA01_00144 / Cj0268c
integral membrane protein	2,626	7,021	80	18,437	41	AA01_00195 / Cj0430
MFS transport protein	2,386	11,875	131	28,331	61	AA01_01136 / Cj0461c
two-component regulator	2,312	181,085	1130	418,741	510	AA01_00264 / Cj0355c
integral membrane protein- carbon starvation	2,183	17,591	345	38,403	147	cstA / Cj0917c
membrane protein insertase YidC	2,139	7,804	115	16,688	48	yidC / Cj0958c
amino acid transporter substrate- binding protein CjaA	2,094	37,948	296	79,479	121	cjaA / Cj0982c
lipoprotein	2,062	40,882	246	84,296	99	AA01_00463 / Cj1074c
protein translocase subunit SecG	1,95	65,424	226	127,556	86	secG / Cj0235c
protein translocase subunit SecA	1,78	24,541	590	43,688	205	secA / Cj0942c
translocation protein TolB	1,749	36,787	413	64,348	141	tolB / Cj0112
multidrug efflux pump protein CmeB	1,567	8,345	242	13,074	74	cmeB / Cj0366c
multidrug efflux pump protein CmeA	1,314	18,631	191	24,489	49	cmeA / Cj0367c*
multidrug efflux pump protein CmeC	-1,086	9,32	128	8,58	23	cmeC / Cj0365c*
**B. Adherence/colonisation**						
lipoprotein CapA	12,809	0,063	0	0,803	2	capA / Cj0628
lipoprotein CapB	5,124	0,128	1	0,656	1	capB / Cj1677
major antigenic peptide Peb3	4,861	33,465	234	162,668	222	peb3 / Cj0289c
fibronectin domain-containing lipoprotein FlpA	2,909	55,849	640	162,49	362	flpA / Cj1279c
transmembrane protein	2,881	23,041	233	66,372	131	AA01_00144 / Cj0268c
major antigenic peptide Peb2	2,443	59,974	411	146,536	196	peb2 / Cj0778
fibronectin/fibrinogen-binding protein FbpA	2,16	8,398	102	18,139	43	fbpA / Cj1349c
lipoprotein	2,132	23,643	137	50,401	57	AA01_01302 / Cj0091
amino acid transporter substrate- binding protein CjaA	2,094	37,948	296	79,479	121	cjaA / Cj0982c
hemolysin tlyA	1,992	2,544	18	5,069	7	tlyA / Cj0588
lipoprotein	1,879	8,755	30	16,448	11	AA01_01303 / Cj0090
lipoprotein JlpA	1,803	12,03	125	21,695	44	jlpA / Cj0983
outer membrane fibronectin-binding protein CadF	1,535	156,487	1395	240,242	418	cadF / Cj1478c
peptidyl-prolyl cis-trans isomerase Peb4	1,333	329,357	2514	438,986	654	peb4 / Cj0596*

bifunctional adhesin/ABC transporter aspartate/glutamate-binding protein Peb1A	1,034	618,663	4481	639,468	904	peb1A / Cj0921c*
**C. Bacterial shape determinant genes**						
penicillin-binding protein PbpC	4,455	4,114	69	18,331	60	pbcC / Cj0652
rod shape-determining protein MreC	2,831	10,913	76	30,898	42	mreC / Cj0277
penicillin-binding protein PbpB	2,661	20,062	337	53,376	175	pbpB / Cj0525c
rod shape-determining protein RodA	2,242	3,13	32	7,016	14	rodA / Cj1282
penicillin-binding protein PbpA	1,688	9,81	176	16,56	58	pbpA / cj0508
rod shape-determining protein MreB	1,533	28,345	274	43,462	82	mreB / Cj0276
**D. Capsule**						
capsular polysaccharide heptosyltransferase HddD	33,303	0,246	4	8,202	26	hddB / Cj1431c
capsule polysaccharide ABC transporter permease KpsM	4,24	7,977	58	33,824	48	kpsM / Cj1448c
capsule polysaccharide ABC transporter ATP-binding protein KpsT	3,897	11,532	71	44,939	54	kpsT / Cj1447c
capsule polysaccharide modification protein KpsC	3,447	2,861	55	9,862	37	kpsC / Cj1414c
capsule polysaccharide ABC transporter permease KpsE	1,882	31,181	324	58,676	119	kpsE / Cj1445c
**E. LOS/Invasion**						
alpha-2,3 sialyltransferase CstIII	20,494	0,122	1	2,494	4	cstIII / Cj1140
sialic acid synthase NeuB1	15,371	0,104	1	1,604	3	neuB1 / Cj1141
glycosyltransferase CgtB	11,528	0,367	4	4,233	9	cgtB / Cj1136
glycosyltransferase	8,966	0,432	4	3,878	7	Cj1137c
beta-1,3 galactosyltransferase WlaN	6,148	0,59	5	3,63	6	wlaN1 / Cj1139c
bifunctional beta-1,4-N-acetylgalactosaminyltransferase/CMP- Neu5Ac synthase NeuA	4,099	0,334	5	1,37	4	neuA / Cj1143
UDP-GlcNAc/Glc 4-epimerase GalE	4,054	27,168	249	110,127	197	galE / Cj1131c
ipid-A-disaccharide synthase LpxB	3,718	22,226	226	82,637	164	lpxB / Cj0288c
heptosyltransferase II WaaF	3,357	3,253	29	10,92	19	waaF / Cj1148
glucosyltransferase	2,417	3,687	53	8,911	25	AA01_00523 / Cj1135
bifunctional D-beta-D-heptose 7-phosphate kinase/D-beta-D-heptose 1- phosphate adenylyltransferase hldE	1,549	6,682	86	10,35	26	hldE / Cj1150c
**F. Iron acquisition**						
enterochelin uptake permease CeuC	6,587	0,803	7	5,288	9	ceuC / Cj1353
iron-uptake ABC transporter ATP- binding protein CfbpC	5,726	2,014	17	11,533	19	ion / Cj0173c
hemin uptake ABC transporter ATP- binding protein ChuC	5,124	1,525	11	7,811	11	chuC / Cj1616
iron-uptake ABC transporter permease CfbpB	4,347	2,198	33	9,554	28	AA01_00463 / Cj0174c
hemin uptake system substrate- binding protein ChuD	3,119	3,069	23	9,572	14	chuD / Cj1617
hemin uptake ABC transporter permease ChuB 132	2,989	1,309	12	3,913	7	chuB / Cj1615
enterochelin uptake ATP-binding protein CeuD	2,846	1,282	9	3,649	5	ceuD / Cj1354
iron transport protein	2,759	1,643	13	4,533	7	Cj0177
ferrous iron transport protein FeoA	2,684	10,051	21	26,975	11	feoA / Cj1397
ferrous iron transport protein FeoB	2,337	6,665	114	15,576	52	feoB1 / Cj1398
enterochelin uptake substrate-binding protein CeuE	2,292	8,242	76	18,892	34	ceuE / Cj1355
iron-uptake ABC transporter substrate-binding protein CfbpA	2,116	59,149	552	125,174	228	cfbpA / Cj0175c

G. Hypothetical proteins

hypothetical protein - capsule	81,977	0,046	1	3,773	16	AA01_00788 / Cj1418c
conserved hypothetical protein	66,606	0,061	1	4,094	13	AA01_00922 / Cj0008
hypothetical protein - LOS/ Invasion	46,112	0,127	1	5,863	9	Cj1145c
pseudogene (IS element)	35,865	0,261	3	9,359	21	Cj0752
hypothetical protein - unknown function	27,326	0,528	3	14,425	16	AA01_00176 / Cj0305c
hypothetical protein - O-linked glycosylation	15,371	0,31	2	4,763	6	Cj1322
hypothetical protein	13,508	2,542	33	34,337	87	AA01_00330 / Cj0030
hypothetical protein - colonisation	10,247	0,434	3	4,444	6	AA01_00111 / Cj0170
hypothetical protein	10,247	0,909	2	9,312	4	Cj0988c
hypothetical protein - Invasion	10,247	0,169	1	1,735	2	AA01_00324 / Cj1555c
hypothetical protein - transport/binding protein	7,416	2,692	38	19,965	55	Cj0046
hypothetical protein - unknown fucntion	6,742	1,243	19	8,38	25	Cj0522
hypothetical protein - chemotaxis?	6,569	6,796	39	44,64	50	AA01_00161 / Cj0286c
hypothetical protein	6,262	0,838	9	5,246	11	Cj0568
hypothetical protein - membrane protein	5,518	0,827	13	4,563	14	Cj0742
hypothetical protein - O-linked glycosylation	5,124	0,296	1	1,517	3	Cj1340c
hypothetical protein	4,919	14,132	50	69,512	48	AA01_01638 / Cj0939c
conserved hypothetical protein	4,747	20,426	136	96,961	126	smc / Cj0706
hypothetical protein - VirK	4,729	1,615	13	7,637	12	AA01_00458 / Cj1069
conserved hypothetical protein	4,315	2,818	19	12,16	16	AA01_01404 / Cj0705
hypothetical protein - Putative cystathionine beta-lyase	3,605	2,5	27	9,014	19	Cj1392
hypothetical protein - DksA	3,586	5,933	20	21,28	14	dksA / Cj0125c
putative ATP-binding protein	3,367	3,83	35	12,897	23	AA01_01034 / Cj1633
hypothetical protein	3,21	5,877	83	18,863	52	AA01_01481 / Cj0621
hypothetical protein	3,151	64,101	200	201,981	123	AA01_00199 / Cj0427
hypothetical protein - motility FliJ	3,054	13,053	52	39,87	31	AA01_01226 / Cj1497c
hypothetical protein - colonisation CfrB	2,996	6,133	118	18,374	69	Cj0444
hypothetical protein	2,966	2,603	19	7,722	11	Cj0380c
hypothetical protein	2,836	17,685	168	50,159	93	AA01_00359 / Cj0760
hypothetical protein - helix-turn-helix domain	2,829	20,853	201	59,002	111	AA01_00375 / Cj1533c
hypothetical protein	2,65	7,654	29	20,285	15	AA01_01177 / Cj1449c
hypothetical protein	2,641	14,04	97	37,08	50	AA01_01040 / Cj1627c
hypothetical protein	2,591	33,01	263	85,528	133	AA01_00048 / Cj0248
hypothetical protein	2,562	3,779	26	9,68	13	AA01_00373 / Cj0984
hypothetical protein - LOS/ Invasion	2,365	5,283	39	12,493	18	AA01_00520 / Cj1132c
hypothetical protein	2,145	4,423	43	9,486	18	AA01_01704 / Cj0881c
hypothetical protein - motility	2,064	365,416	1303	754,35	525	AA01_00198 / Cj0428
hypothetical protein	2,049	8,547	25	17,516	10	AA01_00758 / Cj1384c
hypothetical protein - poly G tract- colonisation	1,971	1,139	13	2,245	5	Cj0617
putative KdpA	1,966	5,589	86	10,988	33	Cj0676
hypothetical protein	1,951	43,12	197	84,109	75	AA01_00381 / Cj0993c

H. Invasion

ATP-dependent protease Lon	3,057	10,56	233	32,278	139	lon / Cj1073c
invasion protein CipA	2,616	3,741	47	9,787	24	cipA / Cj0685c

CorA-like Mg2+ transporter protein	2,562	2,524	18	6,466	9	AA01_00399 / Cj1011
peptidyl-prolyl cis-trans isomerase Ppi	2,515	12,263	55	30,843	27	ppi / Cj1171c
bipartate energy taxis response protein CetB	2,35	47,141	218	110,794	100	cetB / Cj1189c
bipartate energy taxis response protein CetA	2,329	63,521	811	147,934	370	cetA / Cj1190c
lipoprotein	2,242	9,438	112	21,155	49	AA01_01166 / Cj0497
invasion antigen CiaB	2,036	18,624	317	37,927	126	ciaB / Cj0914c
ATP-dependent protease proteolytic subunit ClpP	1,931	46,389	252	89,601	95	clpP / Cj0192c
aspartate aminotransferase AspB	1,524	39,302	427	59,891	127	aspB / Cj0762c
serine protease HtrA	1,437	88,186	1162	126,759	326	htrA / Cj1228c*
ABC transporter ATP-binding protein IamA	1,409	5,958	40	8,395	11	iamA / Cj1647*
ABC transporter permease IamB	1,067	2,328	24	2,485	5	iamB / Cj1646*
paralysed flagellum protein PflA	-1,012	14,149	311	13,986	60	pflA / Cj1565c*
**I. Colonisation**						
two-component sensor histidine kinase DccS signaling colonisation	2,638	12,116	134	31,966	69	dccS / Cj1222c
branched-chain amino acid ABC transporter substrate-binding protein LivK	4,639	23,575	243	109,356	219	livK / Cj1018c
branched-chain amino acid ABC transporter substrate-binding protein LivJ	4,207	26,44	274	111,241	225	livJ / Cj1019c
chaperone protein DnaJ	3,705	6,239	65	23,113	47	dnaJ / Cj1260c
periplasmic protein- fitness in chickens	3,052	5,442	47	16,612	28	AA01_01544 / Cj0561c
sulfoxide reductase catalytic subunit	2,885	55,411	460	159,848	259	AA01_00241 / Cj0379c
cytochrome C551 peroxidase DocA	1,77	55,198	469	97,687	162	docA / Cj0020c
phospholipase A PldA	1,397	3,59	33	5,016	9	pldA / Cj1351*
**J. Regulator/Signal transduction**						
transcriptional regulator	14,678	4,564	37	66,994	106	AA01_01533 / Cj0571
stationary phase survival protein SurE	6,244	4,435	32	27,694	39	surE / Cj0293
type IIS restriction/modification enzyme	3,652	2,712	94	9,903	67	AA01_00302 / Cj0031
S-ribosylhomocysteine lyase LuxS	2,734	119,438	549	326,593	293	luxS / Cj1198
two-component sensor histidine kinase DccS signaling colonisation	2,638	12,116	134	31,966	69	dccS / Cj1222c
guanosine-3',5'-bis(diphosphate) 3'-pyrophosphohydrolase SpoT	2,204	16,526	337	36,432	145	spoT / Cj1272c
two-component sensor histidine kinase RacS	1,733	18,035	207	31,248	70	racS / Cj1262
ferric uptake regulator Fur	1,37	55,207	243	75,662	65	fur / Cj0400*
two-component response regulator CbrR	1,291	56,31	651	72,681	164	cbrR / Cj0643*
two-component regulator DccR	1,228	27,65	171	33,967	41	dccR / Cj1223c*
two-component regulator RacR	1,139	131,247	819	149,433	182	racR / Cj1261*
heat shock transcriptional regulator HspR	-1,641	159,38	555	97,108	66	hspR / Cj1230
transcriptional regulator CmeR	-1,644	27,22	160	16,561	19	cmeR / Cj0368c
**K. Toxin**						
cytolethal distending toxin CdtA	1,23	26,689	200	32,818	48	cdtA / Cj0079c*
cytolethal distending toxin CdtB	-1,025	28,339	210	27,657	40	cdtB / Cj0078c*
cytolethal distending toxin CdtC	-1,561	10,58	56	6,776	7	cdtC / Cj0077c
**L. Chemotaxis**						
MCP protein-glutamate methylesterase CheB	1,868	55,882	288	104,386	105	cheB / Cj0924c
chemotaxis protein, CheW	1,616	69,317	336	112,042	106	cheW / Cj0283c

chemotaxis histidine kinase CheA	1,416	100,231	2150	141,879	594	cheA / Cj0284c*
chemotaxis protein CheV	1,402	120,068	1067	168,351	292	cheV / Cj0285c*
MCP protein methyltransferase CheR	1,325	23,749	174	31,469	45	cheR / Cj0923c*
methyl-accepting chemotaxis signal transduction protein Tlp3	1,164	109,855	888	127,882	231	tlp3 / Cj1564*
chemotaxis protein CheY	1,062	129,611	473	137,587	98	cheY / Cj1118c*
methyl-accepting chemotaxis signal transduction protein DocC	-1,011	3,072	25	3,038	4	docC / Cj0262c*
MCP-domain signal transduction protein DocB	-1,012	34,202	565	33,806	109	docB / Cj0019c*
**M. Chaperones/stress responses**						
chaperone protein DnaJ	3,704718848	6,238705	65	23,11265	47	dnaJ / Cj1260c
chaperone protein HtpG	2,748491869	20,2176	343	55,5679	184	htpG / Cj0518
bacterioferritin Dps	2,063593112	588,2248	2458	1213,857	990	dps / Cj1534c
alkyl hydroperoxide reductase AhpC	1,670290607	1304,181	7230	2178,362	2357	ahpC / Cj0334
superoxide dismutase SodB	1,532397949	445,8644	2745	683,2417	821	sodB / Cj0169
catalase KatA	1,24937839	141,319	1870	176,561	456	katA / Cj1385*
chaperone DnaK	-1,11934616	429,5505	7467	383,7513	1302	dnaK / Cj0759*
chaperone GroEL	-1,15218682	1512,849	23011	1313,024	3898	groEL / Cj1221*
co-chaperonin GroES	-1,6542464	2294,078	5560	1386,782	656	groES / Cj1220
peroxide stress regulator PerR	-1,74033069	56,07198	214	32,21915	24	perR / Cj0322
chaperone protein ClpB	-2,60359699	353,2756	8444	135,6875	633	clpB / Cj0509c
**N. Protein syntheis/modification/secretion**						
tRNA(Ile)-lysidine synthase TilS	9,82	1,338	12	13,137	23	tilS / Cj1453c
elongation factor Tu	9,564	232,969	2596	2228,163	4846	tuf / Cj0470
elongation factor G	6,726	52,755	1017	354,812	1335	fusA1 / Cj0493
SsrA-binding protein	4,845	21,871	92	105,966	87	smpB / Cj1105
signal peptidase I LepP	3,9	8,498	67	33,144	51	AA01_00078 / Cj0856
elongation factor P	3,608	26,828	142	96,799	100	efp / Cj0551
transcription elongation factor NusA	3,481	30,853	312	107,412	212	nusA / Cj0460
elongation factor Ts	3,411	74,099	739	252,758	492	tsf / Cj1181c
chaperone protein HtpG	2,748	20,218	343	55,568	184	htpG / Cj0518
selenocysteine-specific elongation factor SelB	2,657	3,22	54	8,554	28	selB / Cj1379
elongation factor EF-4	2,281	9,828	164	22,414	73	lepA / Cj1030c
Sec-independent protein translocase TatC	2,264	12,549	86	28,41	38	tatC / Cj0578c
heat-inducible transcription repressor HrcA	1,841	145,483	1074	267,895	386	hrcA / Cj0757
**O. O-linked glycosylation**						
imidazole glycerol phosphate synthase subunit HisH	17,932	0,355	2	6,373	7	hisH1 / Cj1315c
3-oxoacyl-ACP synthase FabH2	8,539	0,304	3	2,598	5	fabH2 / Cj1303
N-acetyltransferase	7,319	0,952	7	6,967	10	AA01_00685 / Cj1298
**P. N-linked glycolisation**						
UDP-N-acetylbacillosamine transaminase PglE	2,868	34,969	377	100,276	211	pglE / Cj1121c
integral membrane protein WlaJ	2,415	11,526	70	27,841	33	wlaJ / Cj1122c
protein glycosylation PglK	2,379	5,337	84	12,695	39	pglK / Cj1130c
glycoprotein CpgA	2,242	10,395	64	23,302	28	cpgA / Cj1670c
GalNAc(5)-diNAcBac-PP- undecaprenol beta-1,3-glucosyltransferase pglI WlaD	2,005	5,327	46	10,679	18	pglI / Cj1128c
UDP-N-acetyl-alpha-D-glucosamine C6 dehydratase PglF	1,612	12,937	213	20,85	67	pglF / Cj1120c

N, N'-diacetylbacillosaminyl- diphospho-undecaprenol alpha-1,3-N-acetylgalactosaminyltransferase PglA	1,601	6,094	64	9,757	20	pglA / Cj1125c
**Q. Colonisation/ Respiration**						
NADH-quinone oxidoreductase subunit I NuoI	2,049	11,742	70	24,064	28	nuoI / Cj1571c
NADH-quinone oxidoreductase I subunit M NuoM	1,971	4,704	65	9,27	25	nuoM / Cj1567c
NADH-quinone oxidoreductase I subunit K NuoK	1,708	4,351	12	7,431	4	nuoK / Cj1569c
NADH-quinone oxidoreductase subunit J NuoJ	1,708	6,847	33	11,694	11	nuoJ / Cj1570c
NADH-quinone oxidoreductase subunit L NuoL	1,667	4,991	83	8,318	27	nouL / Cj1568c
NADH-quinone oxidoreductase subunit G NuoG	1,574	13,379	306	21,058	94	nuoG / Cj1573c
formate dehydrogenase iron-sulfur subunit FdhA	1,559	34,722	207	54,144	63	fdhA / Cj1510c
NADH-quinone oxidoreductase subunit C NuoC	1,464	13,275	98	19,433	28	nuoC / Cj1577c*
**R. Energy metabolism**						
thiredoxin	9,678	3,964	18	38,363	34	AA01_01003 / Cj1664
cytochrome C	5,903	22,776	92	134,45	106	AA01_00409 / Cj1020c
thioredoxin	2,622	319,992	936	839,015	479	trxA / Cj0147c
nitrogen fixation protein NifU	2,738	64,481	582	176,538	311	nfiU / Cj0239c
Ni/Fe-hydrogenase small subunit HydA2	2,339	17,372	241	40,624	110	AA01_00313 / Cj1399c
**S. Mucin degradation**						
sulfatase family protein	1,988	9,376	134	18,643	52	AA01_00040 / Cj0256
tRNA N6-adenosine threonylcarbamoyltransferase	1,708	7,051	66	12,042	22	AA01_00306 / Cj1344c
**T. Periplasmic proteins**						
periplasmic protein	12,484	18,336	71	228,905	173	AA01_01000 / Cj1668c
periplasmic protein	5,623	8,947	82	50,312	90	AA01_01030 / Cj1637c
periplasmic protein	4,01	8,121	69	32,562	54	dmsD / Cj0784
periplasmic protein	2,835	252,558	394	715,965	218	AA01_00114 / Cj0168c
periplasmic protein	2,8	76,488	505	214,183	276	AA01_00754 / Cj1380
quinol dehydrogenase periplasmic subunit	2,277	35,315	243	80,417	108	napG / Cj0781
periplasmic protein	2,255	88,266	359	199,034	158	AA01_01001 / Cj1666c
**U. Flagellar proteins**						
flagellar biosynthesis protein FliR	7,173	2,804	20	20,116	28	fliR / Cj1179c
flagellar biosynthesis protein FlhB	6,904	37,281	377	257,383	508	flhB / Cj0335
motility protein	5,124	0,296	1	1,517	3	Cj1340c
flagellar motor protein MotB	4,534	23,883	165	108,274	146	motB / Cj0336c
sigma54 associated transcriptional activator FlgR	4,262	19,189	232	81,788	193	flgR / Cj1024c
flagellar biosynthesis protein FlhA	3,697	8,714	176	32,217	127	flhA / Cj0882c
flagellin modification protein ptmA	3,416	0,838	6	2,863	4	ptmA / Cj1332
MotA/TolQ/ExbB proton channel family protein ExbB3	3,24	13,194	68	42,748	43	exbB3 / Cj0109
flagellar basal body-associated protein FliL	2,59	54,346	271	140,764	137	fliL / Cj1408
flagellar motor protein MotA	2,586	43,381	313	112,197	158	motA / Cj0337c
flagellum-specific ATP synthase FliI	2,508	7,459	96	18,71	47	fliI / Cj0195
flagellar motor switch protein FliY	2,189	27,21	213	59,561	91	fliY / Cj0059c
RNA polymerase factor sigma-54	2,021	6,112	71	12,349	28	rpoN / Cj0670
flagellar biosynthesis protein FliP	1,983	13,626	93	27,025	36	fliP / Cj0820c

flagellar basal body P-ring biosynthesis protein FlgA	1,59	9,421	58	14,98	18	flgA / Cj0769c
flagellar protein FliS	1,556	68,732	247	106,929	75	fliS / Cj0549
flagellar MS-ring protein fliF	1,438	21,883	342	31,473	96	fliF / Cj0318*
flagellar biosynthesis regulator FlhF	1,33	15,395	208	20,477	54	flhF / Cj0064c*
flagellar biosynthesis protein FliQ	1,25	32,706	82	40,871	20	fliQ / Cj1675*
flagellar biosynthesis RNA polymerase sigma factor FliA	1,233	121,057	806	149,289	194	fliA / Cj0061c*
flagellar hook-basal body protein FliE	1,203	466,293	1286	561,042	302	fliE / Cj0526c*
flagellar motor switch protein fliN	1,156	46,352	133	53,568	30	fliN / Cj0351*
flagellar motor switch protein FliM	1,141	43,874	440	50,066	98	fliM / Cj0060c*
flagellar basal body rod protein FlgB	1,04	407,825	1636	424,032	332	flgB / Cj0528c*
flagellar basal body rod protein FlgC	1,022	740,557	3404	756,849	679	flgC / Cj0527c*
signal transduction histidine kinase flgS	1,009	33,785	320	34,079	63	flgS / Cj0793*
flagellin FlaB	-1,009	364,792	4019	361,416	757	flaB / Cj1338c*
flagellar assembly protein FliH	-1,038	13,089	101	12,615	19	fliH / Cj0320*
flagellar assembly protein FliW	-1,041	216,484	784	207,968	147	fliW / Cj1075*
ATP-binding protein FlhG	-1,093	13,911	112	12,728	20	flhG / Cj0063c*
flagellin C FlaC	-1,101	540,458	3764	490,692	667	flaC / Cj0720c*
flagellar biosynthesis protein FlgM	-1,133	1379,842	2537	1217,758	437	flgM / Cj1464*
flagellar basal body rod protein FlgG	-1,188	541,168	3980	455,614	654	flgG / Cj0698*
flagellar basal-body rod protein FlgG2	-1,266	983,777	7427	777,069	1145	flgG2 / Cj0697*
flagellar hook-length control protein FliK	-1,35	106,132	1771	78,603	256	fliK / Cj0041*
flagellar hook-associated protein FliD	-1,354	546,766	9793	403,875	1412	fliD / Cj0548*
flagellar hook protein FlgE	-1,423	197,928	4775	139,106	655	flgE / Cj1729c*
flagellar hook-associated protein FlgK	-1,507	290,768	4933	192,978	639	flgK / Cj1466
flagellar basal body P-ring protein FlgI	-1,52	551,099	5358	362,565	688	flgI / Cj1462
flagellin FlgL	-1,657	377,463	7897	227,754	930	flgL / Cj0887c
flagellar hook protein FlgE	-1,75	221,033	3362	126,317	375	flgE / Cj0043
RNA polymerase sigma factor RpoD	-1,794	113,855	1976	63,471	215	rpoD / Cj1001
flagellin A FlaA	-1,875	1076,082	11953	573,9	1209	flaA / Cj1339c
flagellar hook assembly protein	-1,932	887,679	7295	459,482	737	flgD / Cj0042
flagellar basal body L-ring protein FlgI	-1,951	938,086	6089	480,712	609	Cj0687c
flagellar motor switch protein fliG	-1,981	28,675	274	14,477	27	fliG / Cj0319
flagellar biosynthesis protein FlgJ	-2,177	161,534	513	74,212	46	flgJ / Cj1463
flagellar protein FlaG	-2,247	1940,768	6596	863,81	573	flaG / Cj0547
**V. Other, protein coding ORFs**						
NOL1/NOP2/sun family protein	6,404	1,538	12	9,853	15	AA01_01467 / Cj0636
ATP-dependent protease ATP-binding subunit ClpX	3,621	11,422	133	41,359	94	Cj0275
DNA methylase	2,365	8,187	52	19,36	24	AA01_01189 / Cj1461
CRISPR-associated endoribonuclease Cas2	2,074	10,47	42	21,713	17	Cj1521c
D-3-phosphoglycerate dehydrogenase SerA	1,508	18,016	265	27,17	78	Cj0891c

[1] Main functional categories and specific functions are as indicated in the 2006 re-annotation of *C. jejuni* NCTC 11168 genome, GenBank accession AL11168.

Supplementary Table 3. Summarised data of the 190 clinical and 2 reference *C. jejuni* strains. Column A represents the number or the name of the strains, column B shows the clinical symptoms, and from columns C to S show the presence or absence of the tested genes. Columns T to V show the extracellular matrix protein binding ability in percentage (%) and column W and X show the adhesion and invasion ability in percentage (%). In the case of the adhesion, the given adhesion values represent the proportion of the adhered bacteria from the total bacteria which was inoculated to the epithelial cells. The invasion values give the rate of the invaded bacteria from the adhered bacterial number (Backert and Hofreuter, 2013). *Pseudomonas aeruginosa* was used as a positive control, binding collagen type IV with 0.16%, fibronectin with 0.16%, and laminin with 0.1 %.

A	B	C	D	E	F	G	H	I	J	K	L	M	N	O	P	Q	R	S	T	U	V	W	X
**Strain no.**	Clinical symptoms	cst-II	cst-III	cadF	iamA	ciaB	cgtB	docA	docB	docC	flgE2	cdtB	flaB	flhB	flgB	wlaN	virB11	hcp	Collagen IV	Fibronectin	Laminin	Adhesion	Invasion
**1**	diarrhea	X	X	✔	✔	✔	✔	✔	✔	X	✔	✔	✔	✔	✔	X	X	X	0,007	0,007	0,011	1,361	15,5
**2**	stomach ache	X	✔	✔	✔	✔	✔	✔	✔	X	✔	✔	✔	✔	✔	✔	X	✔	0,007	0,011	0,007	0,118	85,6
**3**	diarrhea	✔	X	✔	✔	✔	✔	✔	✔	✔	✔	✔	✔	✔	✔	X	X	X	0,001	0,002	0,003	1,897	2,2
**4**	diarrhea	✔	X	✔	✔	✔	✔	✔	✔	✔	✔	✔	✔	✔	✔	X	X	X	0,005	0,008	0,016	1,501	2,8
**5**	diarrhea	X	X	✔	✔	X	X	X	X	X	✔	✔	✔	X	✔	X	X	X	0,001	0,001	0,002	0,538	19,1
**6**	unknown	X	X	✔	✔	✔	✔	✔	✔	X	✔	✔	✔	✔	✔	X	X	✔	0,003	0,004	0,01	1,926	1,9
**7**	colitis	X	X	✔	✔	✔	✔	✔	✔	X	✔	✔	✔	✔	✔	X	X	✔	0,03	0,023	0,042	0,415	20,7
**8**	unknown	X	✔	✔	✔	X	✔	✔	✔	✔	✔	✔	✔	✔	✔	✔	X	X	0,003	0,004	0,005	0,176	73,7
**9**	colitis	✔	X	✔	✔	X	✔	✔	✔	X	✔	✔	✔	✔	✔	X	X	✔	0,029	0,001	0,001	1,174	13,2
**10**	diarrhea	✔	X	✔	✔	✔	✔	✔	✔	✔	✔	✔	✔	✔	✔	X	X	X	0,005	0,019	0,029	0,205	72,8
**11**	diarrhea	✔	X	✔	✔	✔	✔	✔	✔	✔	✔	✔	✔	✔	✔	X	X	X	0,012	0,025	0,015	0,412	98,5
**12**	unknown	X	X	✔	✔	✔	✔	✔	✔	✔	✔	✔	✔	✔	✔	X	X	✔	0	0	0	0,146	21,5
**14**	gastroenteritis	X	X	✔	✔	✔	X	✔	✔	X	✔	✔	✔	✔	✔	X	X	X	0,006	0,007	0,003	0,081	52,7
**16**	unknown	✔	X	✔	✔	✔	X	✔	✔	X	✔	✔	✔	✔	✔	X	X	✔	0,001	0,004	0	0,422	14,8
**17**	unknown	X	X	✔	✔	X	X	X	X	X	✔	✔	✔	✔	✔	X	X	✔	0,009	0,033	0,006	0,622	28,8
**18**	diarrhea	✔	X	✔	✔	✔	✔	✔	✔	X	✔	✔	✔	X	✔	X	X	X	0,014	0,007	0,003	0,757	105,3
**21**	diarrhea	✔	X	✔	✔	✔	✔	✔	✔	✔	✔	✔	✔	✔	✔	X	X	X	0	0	0	0,143	148,5
**22**	diarrhea	✔	X	✔	✔	✔	✔	✔	✔	✔	✔	✔	✔	✔	✔	X	X	X	0,004	0,003	0,002	1,796	20
**23**	diarrhea	✔	X	✔	✔	✔	✔	✔	✔	✔	✔	✔	✔	✔	✔	X	X	X	0,004	0,004	0,001	0,014	126,6
**24**	diarrhea	✔	X	✔	✔	✔	✔	✔	✔	✔	✔	✔	✔	✔	✔	X	X	X	0,016	0,002	0,063	0,295	15,9
**26**	stomach ache	X	X	✔	✔	✔	✔	✔	✔	✔	✔	✔	✔	✔	✔	X	X	✔	0	0	0	0,098	0,12
**25**	diarrhea	✔	X	✔	✔	✔	X	✔	✔	X	✔	✔	✔	✔	✔	X	X	X	0,003	0,006	0,014	0,643	27,2
**27**	unknown	✔	X	✔	✔	✔	✔	✔	✔	✔	✔	✔	✔	✔	✔	X	X	X	0,01	0,004	0,002	0,729	9,1
**28**	gastroenteritis	✔	X	✔	✔	✔	✔	✔	✔	✔	✔	✔	✔	✔	✔	X	X	X	0,001	0,014	0,003	0,073	54,6
**29**	colitis ulcerosa	✔	X	✔	✔	✔	✔	✔	✔	✔	✔	✔	✔	✔	✔	X	X	X	0,005	0,017	0,001	0,705	113
**30**	gastroenteritis	X	X	✔	✔	✔	✔	✔	✔	X	✔	✔	✔	✔	✔	X	X	X	0	0,001	0,008	0,105	10
**31**	bloody diarrhea	X	X	✔	✔	✔	X	✔	✔	✔	✔	✔	✔	✔	✔	X	X	✔	0,107	0,027	0,013	1,04	25,5
**32**	diarrhea	✔	X	✔	✔	✔	✔	✔	✔	✔	✔	✔	✔	✔	✔	X	X	X	0,165	0,092	0,002	0,283	13,8
**33**	diarrhea	X	X	✔	✔	✔	X	✔	✔	✔	✔	✔	✔	✔	✔	X	X	✔	0,169	0,03	0,062	0,192	43,5
**34**	diarrhea	✔	X	✔	✔	✔	X	✔	✔	✔	✔	✔	✔	✔	✔	X	X	X	0,21	0,039	0,181	0,333	101,8
**35**	diarrhea	X	X	✔	✔	✔	✔	✔	✔	✔	✔	✔	✔	✔	✔	X	X	X	0,036	0,008	0,034	6,637	51,6
**36**	diarrhea	X	X	✔	✔	X	X	X	X	✔	✔	✔	✔	X	✔	X	X	X	0,142	0,114	0,117	1,291	9,5

**38**	diarrhea	X	X	✔	✔	✔	✔	✔	✔	✔	✔	✔	✔	✔	✔	X	X	X	1,433	0,237	0,227	0,159	191,3
**39**	bloody diarrhea	✔	X	✔	✔	✔	X	✔	✔	X	✔	✔	✔	✔	✔	X	X	✔	0	0	0,012	0,011	3,2
**40**	diarrhea	✔	X	✔	✔	✔	X	✔	✔	X	✔	✔	X	✔	✔	X	X	X	0,014	0,012	0,006	1,574	9,2
**41**	diarrhea	X	X	✔	✔	X	X	✔	X	X	✔	✔	✔	✔	✔	X	X	X	0,001	0,033	0,003	0,308	3,7
**42**	diarrhea	X	✔	✔	✔	✔	X	✔	✔	✔	✔	✔	✔	✔	✔	✔	X	X	0,014	0,03	0,004	0,023	43,5
**43**	bloody diarrhea	✔	X	✔	✔	✔	✔	✔	✔	X	✔	✔	✔	✔	✔	X	X	X	0,081	0,011	0,011	0,119	11,9
**44**	diarrhea	X	X	✔	✔	✔	X	✔	✔	X	✔	✔	✔	✔	✔	✔	X	X	0,062	0,566	0,71	0,03	38,5
**45**	diarrhea	X	X	✔	✔	✔	X	✔	✔	X	✔	✔	✔	✔	✔	✔	X	✔	0,178	0,071	0,012	0,246	28
**46**	diarrhea	✔	X	✔	✔	✔	X	✔	✔	X	✔	✔	✔	✔	✔	✔	X	X	0	0	0	0,201	8,3
**47**	diarrhea	X	X	✔	✔	✔	X	✔	✔	X	✔	✔	✔	✔	✔	✔	X	X	0,784	0,677	0,694	0,287	22,1
**48**	diarrhea	X	X	✔	✔	✔	✔	✔	✔	✔	✔	✔	✔	✔	✔	X	X	X	0,152	0,034	0,008	1,616	3,8
**49**	diarrhea	✔	X	✔	✔	✔	✔	✔	✔	✔	✔	✔	✔	✔	✔	X	X	X	0,247	0,254	0,254	3,574	28,1
**50**	diarrhea	✔	X	✔	✔	✔	✔	✔	✔	✔	✔	✔	✔	✔	✔	✔	X	X	0,008	0,008	0,003	0,462	6,5
**51**	diarrhea	X	X	✔	✔	✔	X	✔	✔	✔	✔	✔	✔	✔	✔	✔	X	X	0,015	0,011	0,075	0,123	241,3
**52**	diarrhea	X	X	✔	✔	X	X	X	X	X	✔	✔	✔	X	✔	✔	X	X	0,004	0,003	0,005	0,217	20
**53**	diarrhea	✔	X	✔	✔	✔	✔	✔	✔	X	✔	✔	✔	✔	✔	✔	X	X	0,005	0,027	0,032	0,06	37,2
**54**	diarrhea	X	X	✔	✔	✔	✔	✔	✔	X	✔	✔	✔	✔	✔	X	X	X	0,528	0,533	1,109	0,474	86,7
**55**	diarrhea	✔	X	✔	✔	✔	✔	✔	✔	✔	✔	✔	✔	✔	✔	X	X	X	0,334	0,468	0,312	0,027	75,5
**56**	diarrhea	✔	X	✔	✔	✔	X	✔	✔	✔	✔	✔	✔	✔	✔	✔	X	✔	0,019	0,035	0,076	0,087	52,4
**57**	diarrhea	X	X	✔	✔	✔	X	✔	✔	X	✔	✔	✔	✔	✔	✔	X	X	0,007	0,003	0,003	2,808	0,6
**58**	diarrhea	X	X	✔	✔	X	X	X	X	X	✔	✔	✔	✔	✔	✔	X	X	0,007	0,023	0,012	0,153	2,6
**59**	diarrhea	X	X	✔	✔	✔	X	✔	✔	X	✔	✔	X	✔	✔	✔	X	X	0,003	0,003	0,002	1,144	7,2
**60**	bloody diarrhea	✔	X	✔	✔	✔	✔	✔	✔	X	✔	✔	✔	✔	✔	✔	X	X	0,001	0,003	0,002	0,261	4,3
**61**	diarrhea	X	X	✔	✔	✔	X	✔	✔	X	✔	✔	✔	✔	✔	✔	X	X	0,616	0,179	0,138	0,192	55,3
**62**	diarrhea	X	X	✔	✔	✔	✔	✔	✔	X	✔	✔	✔	✔	✔	✔	X	X	0,003	0,006	0,012	0,376	11,5
**63**	bloody diarrhea	X	X	✔	✔	✔	X	✔	✔	✔	✔	✔	✔	✔	✔	X	X	X	0,124	0,026	0,016	0,489	5,2
**64**	diarrhea	✔	X	✔	✔	✔	✔	✔	✔	✔	✔	✔	✔	✔	✔	X	X	✔	0,01	0,009	0,013	0,293	25,6
**65**	diarrhea	✔	X	✔	✔	✔	X	✔	✔	✔	✔	✔	✔	✔	✔	X	X	X	0,007	0,029	0,016	0,397	11,5
**66**	diarrhea	✔	X	✔	✔	✔	✔	✔	✔	✔	✔	✔	✔	✔	✔	X	X	✔	0,012	0,041	0,103	0,147	40,4
**67**	diarrhea	✔	X	✔	✔	✔	✔	✔	✔	✔	✔	✔	✔	✔	✔	X	X	X	0,117	0,008	0,009	0,23	23,1
**68**	diarrhea	X	✔	✔	✔	✔	✔	✔	✔	✔	✔	✔	✔	✔	✔	X	X	X	0,008	0,016	0,024	0,1	56,1
**69**	diarrhea	✔	X	✔	✔	✔	✔	✔	✔	✔	✔	✔	✔	✔	✔	X	X	X	0,106	0,014	0,007	0,584	13,1
**70**	diarrhea	X	X	✔	✔	✔	X	✔	✔	✔	✔	✔	✔	✔	✔	✔	X	✔	0,001	0,003	0	0,111	63,1
**71**	diarrhea	X	X	✔	✔	✔	✔	✔	✔	✔	✔	✔	✔	✔	✔	✔	X	X	0,252	0,33	0,111	0,147	32,1
**72**	diarrhea	X	X	✔	✔	✔	✔	✔	✔	✔	✔	✔	X	✔	✔	X	X	X	0,497	4,334	7,001	0,113	208,4
**73**	diarrhea	X	✔	✔	✔	✔	✔	✔	✔	✔	✔	✔	✔	✔	✔	X	X	X	0,007	0,016	0,008	1,231	3,4
**74**	diarrhea	X	X	✔	✔	✔	✔	✔	✔	X	✔	✔	✔	✔	✔	X	X	✔	0,008	0,02	0,008	0,4	23,1
**75**	diarrhea	✔	X	✔	✔	✔	X	✔	✔	✔	✔	✔	✔	✔	✔	X	X	✔	0,112	0,519	0,441	0,24	3,1
**76**	diarrhea	✔	X	✔	✔	✔	X	✔	✔	✔	✔	✔	✔	✔	✔	✔	X	X	0,102	0,034	0,034	0,075	66,1
**77**	diarrhea	X	X	✔	✔	✔	✔	✔	✔	✔	✔	✔	✔	✔	✔	✔	X	X	0,039	0,027	0,026	0,222	31,9
**78**	diarrhea	✔	X	✔	✔	✔	✔	✔	✔	✔	✔	✔	✔	✔	✔	X	X	X	0,007	0,024	0,133	0,18	10,4
**79**	bloody diarrhea	X	X	✔	✔	✔	✔	✔	✔	X	✔	✔	✔	✔	✔	X	X	✔	0,081	0,074	0,063	2,571	58,6
**80**	diarrhea	✔	X	✔	✔	✔	✔	✔	✔	X	✔	✔	✔	✔	✔	X	X	✔	0,15	0,815	2,967	0,348	295,5
**81**	diarrhea	X	X	✔	✔	✔	✔	✔	✔	X	✔	✔	X	✔	✔	X	X	X	0,076	0,883	0,588	1,11	0,4
**82**	diarrhea	✔	X	✔	✔	✔	✔	✔	✔	X	✔	✔	✔	✔	✔	✔	X	✔	0,009	0,01	0,008	0,62	63,9
**84**	bloody diarrhea	X	X	✔	✔	✔	✔	✔	✔	X	✔	✔	✔	✔	✔	X	X	✔	0,132	0,659	0,814	0,282	275,4
**85**	diarrhea	✔	X	✔	✔	✔	✔	✔	✔	X	✔	✔	✔	✔	✔	X	X	✔	1,133	2,667	2,8	0,082	99
**86**	diarrhea	✔	X	✔	✔	✔	✔	✔	✔	✔	✔	✔	✔	✔	✔	X	X	X	0,005	0,026	0,015	0,97	145,9
**87**	bloody diarrhea	X	✔	✔	✔	✔	✔	✔	✔	✔	✔	✔	✔	✔	✔	✔	X	X	0,003	0,006	0,002	0,399	81
**88**	diarrhea	X	✔	✔	✔	✔	✔	✔	✔	✔	✔	✔	✔	✔	✔	✔	X	✔	0,223	0,329	0,152	0	10

**89**	diarrhea	X	X	✔	✔	✔	X	✔	✔	✔	✔	✔	✔	✔	✔	✔	X	X	0,027	0,01	0,007	0,34	9,2
**90**	diarrhea	X	X	✔	✔	✔	✔	✔	✔	✔	✔	✔	✔	✔	✔	X	X	X	0,002	0,007	0,001	0,092	18,9
**91**	diarrhea	✔	X	✔	✔	✔	✔	✔	✔	✔	✔	✔	✔	✔	✔	✔	X	X	0,025	0,003	0,007	0,226	53,1
**92**	diarrhea	✔	X	✔	✔	✔	✔	✔	✔	✔	✔	✔	✔	✔	✔	✔	X	✔	0,341	0,055	0,007	0,523	126,6
**94**	diarrhea	X	X	✔	✔	✔	X	✔	✔	X	✔	✔	✔	✔	✔	X	X	✔	0,251	0,013	0,006	0,168	59,9
**95**	diarrhea	X	✔	✔	✔	✔	X	✔	✔	X	✔	✔	✔	✔	✔	✔	X	X	0,149	0,241	0,089	1,606	26,1
**96**	diarrhea	X	X	✔	✔	✔	✔	✔	✔	X	✔	✔	✔	✔	✔	✔	X	✔	0,235	0,151	0,012	0,102	92,7
**97**	diarrhea	X	X	✔	✔	✔	✔	✔	✔	X	✔	✔	✔	✔	✔	✔	X	✔	0,001	0,001	0,017	0,468	33,6
**98**	bloody diarrhea	X	X	✔	✔	✔	✔	✔	✔	✔	✔	✔	✔	✔	✔	X	X	X	0,021	0,014	0,009	0,135	26
**99**	diarrhea	✔	X	✔	✔	✔	✔	✔	✔	✔	✔	✔	✔	✔	✔	X	X	X	0,003	0,014	0,002	0,157	107,1
**100**	bloody diarrhea	X	X	✔	✔	✔	✔	✔	✔	X	✔	✔	✔	✔	✔	✔	X	X	1,256	3,222	0,996	2,418	6,8
**101**	diarrhea	X	✔	✔	✔	✔	✔	✔	✔	✔	✔	✔	✔	✔	✔	✔	X	X	8,239	12,802	0,264	0,111	23,3
**102**	diarrhea	X	✔	✔	✔	✔	✔	✔	✔	✔	✔	✔	✔	✔	✔	✔	X	X	0,015	0,073	0,007	0,445	129,2
**103**	diarrhea	X	✔	✔	✔	✔	✔	✔	✔	✔	✔	✔	✔	✔	✔	✔	X	X	0,006	0,006	0,003	1,156	43,8
**104**	diarrhea	X	✔	✔	✔	✔	✔	✔	✔	✔	✔	✔	✔	✔	✔	✔	X	X	0,004	0,016	0,015	0,598	117,5
**105**	diarrhea	X	✔	✔	✔	✔	✔	✔	✔	✔	✔	✔	✔	✔	✔	✔	X	X	0,01	0,016	0,01	0,762	164,7
**106**	diarrhea	X	X	✔	✔	X	X	X	X	X	✔	✔	✔	✔	✔	X	X	X	0,019	0,024	0,008	0,896	15,6
**107**	diarrhea	X	X	✔	✔	X	X	X	X	X	✔	✔	✔	✔	✔	✔	X	X	0,005	0,051	0,062	0,373	13,1
**108**	diarrhea	X	X	✔	✔	X		X		X	✔	✔	✔	✔	✔	X	X	X	0,001	0,006	0,005	0,242	53,3
**109**	diarrhea	X	X	✔	✔	✔	✔	✔	✔	X	✔	✔	✔	✔	✔	✔	X	✔	0,004	0,002	0,002	1,85	210,7
**110**	bloody diarrhea	✔	X	✔	✔	✔	✔	✔	✔	X	✔	✔	✔	✔	✔	X	X	✔	0,011	0,032	0,009	0,15	247,2
**111**	bloody diarrhea	X	✔	✔	✔	✔	✔	✔	✔	✔	✔	✔	✔	✔	✔	✔	X	✔	0,006	0,006	0,006	0,469	30,5
**112**	diarrhea	X	✔	✔	✔	✔	X	✔	✔	✔	✔	✔	✔	✔	✔	✔	X	X	0,001	0	0	1,859	33,8
**113**	diarrhea	X	✔	✔	✔	✔	X	✔	✔	X	✔	✔	✔	✔	✔	✔	X	X	0,001	0,001	0,001	0,073	71
**114**	diarrhea	X	X	✔	✔	✔	X	✔	✔	X	✔	✔	✔	✔	✔	✔	X	X	0,023	0,005	0,001	0,082	111,4
**115**	diarrhea	X	✔	✔	✔	✔	X	✔	✔	✔	✔	✔	✔	✔	✔	✔	X	X	0,001	0,002	0,001	0,782	55,8
**116**	diarrhea	✔	X	✔	✔	✔	X	✔	✔	X	✔	✔	✔	✔	✔	✔	X	X	0,005	0,006	0,005	0,84	3,1
**117**	diarrhea	X	✔	✔	✔	✔	X	✔	✔	✔	✔	✔	✔	✔	✔	✔	X	X	0,001	0,001	0,001	1,236	23,7
**118**	diarrhea	X	✔	✔	✔	✔	X	✔	✔	✔	✔	✔	✔	✔	✔	✔	X	X	0,245	0,099	0,268	0,889	38,8
**119**	bloody diarrhea	X	X	✔	✔	✔	✔	✔	✔	✔	✔	✔	✔	✔	✔	✔	X	X	0,105	0,109	0,13	2,456	20
**120**	diarrhea	X	✔	✔	✔	✔	X	✔	✔	✔	✔	✔	✔	✔	✔	✔	X	X	0,002	0,003	0,001	1,887	6,5
**121**	bloody diarrhea	X	✔	✔	✔	✔	X	✔	✔	✔	✔	✔	✔	✔	✔	✔	X	X	0,001	0,001	0,002	0,363	9,9
**122**	diarrhea	X	X	✔	✔	✔	X	✔	✔	✔	✔	✔	✔	✔	✔	✔	X	X	0	0	0,001	0,809	13,4
**123**	diarrhea	X	X	✔	✔	✔	✔	✔	✔	X	✔	✔	✔	✔	✔	✔	X	✔	0,014	0,013	0,004	9,316	28,5
**124**	diarrhea	X	X	✔	✔	✔	X	✔	✔	✔	✔	✔	✔	✔	✔	X	X	X	0,181	0,208	0,243	0,289	24,3
**125**	diarrhea	X	X	✔	✔	✔	X	✔	✔	X	✔	✔	✔	✔	✔	✔	X	X	0,035	0,019	0,006	5,443	3,6
**126**	bloody diarrhea	X	X	✔	✔	✔	X	✔	✔	X	✔	✔	✔	✔	✔	✔	X	X	0,028	0,115	0,028	1,235	0,2
**127**	diarrhea	✔	X	✔	✔	✔	X	✔	✔	X	✔	✔	✔	✔	✔	✔	X	✔	0,055	0,085	0,043	0,109	51,9
**128**	diarrhea	✔	X	✔	✔	✔	✔	✔	✔	X	✔	✔	✔	✔	✔	✔	X	✔	0,014	0,019	0,013	0,53	31,5
**129**	diarrhea	X	X	✔	✔	✔	✔	✔	✔	X	✔	✔	✔	✔	✔	✔	X	✔	0,184	0,011	0,021	0,187	20,6
**130**	diarrhea	✔	X	✔	✔	✔	X	✔	✔	X	✔	✔	✔	✔	✔	✔	X	✔	0	0	0	0	0
**131**	diarrhea	X	X	✔	✔	✔	X	✔	✔	✔	✔	✔	✔	✔	✔	✔	X	✔	0,02	0,071	0,017	1,951	8,1
**132**	diarrhea	X	X		X	X	X	X	X	X	✔	✔	✔	✔	✔	✔	X	X	0,048	0,007	0,003	0,766	34,3
**133**	diarrhea	X	✔	✔	✔	✔	✔	✔	✔	✔	✔	✔	✔	✔	✔	✔	X	X	0,005	0,022	0,007	0,981	42,4
**134**	bloody diarrhea	X	X	✔	✔	X	✔	X	X	X	✔	✔	✔	✔	✔	✔	X	✔	0,008	0,011	0,001	1,081	23
**135**	diarrhea	X	X	✔	✔	✔	✔	✔	✔	X	✔	✔	✔	✔	✔	X	X	X	0,023	0,042	0,007	15,537	0,2
**136**	diarrhea	✔	X	✔	✔	✔	X	✔	✔		✔	✔	✔	✔	✔	X	X	✔	0,023	0,019	0,001	4,006	14,4
**138**	bloody diarrhea	X	X	✔	✔	✔	✔	✔	✔	X	✔	✔	✔	✔	✔	✔	X	X	0,005	0,024	0,002	1,925	6,1
**139**	diarrhea	X	X	✔	✔	✔	X	✔	✔	X	✔	✔	✔	✔	✔	✔	X	X	0,131	0,413	0,462	0,226	15,7

**140**	diarrhea	✔	X	✔	✔	✔	✔	✔	✔		✔	✔	✔	✔	✔	✔	X	X	0,006	0,012	0	6,962	7,6
**141**	unknown	✔	X	✔	✔	X	X	✔	✔	X	✔	✔	✔	✔	✔	✔	X	X	0,005	0,008	0,003	0,514	57,1
**142**	bloody diarrhea	X	✔	✔	✔	✔	✔	✔	✔	X	✔	✔	X	✔	✔	X	X	✔	0,002	0,006	0,001	0,39	12,1
**143**	diarrhea	✔	X	✔	✔	✔	✔	✔	✔	X	✔	✔	✔	✔	✔	✔	X	X	0,001	0,01	0,001	2,239	17,9
**144**	diarrhea	X	X	✔	✔	✔	✔	✔	✔	X	✔	✔	✔	✔	✔	✔	X	✔	0,007	0,005	0,006	6,186	5,7
**145**	unknown	X	X	✔	✔	✔	✔	✔	✔	X	✔	✔	✔	✔	✔	✔	X	X	0,003	0,013	0,009	0,221	68,9
**146**	diarrhea	X	X	✔	✔	✔	✔	✔	✔	✔	✔	✔	✔	✔	✔	✔	X	X	0,105	0,092	0,128	0,328	15,7
**147**	diarrhea	X	X	✔	✔	X	✔	X	✔	X	✔	✔	✔	✔	✔	✔	X	X	0,176	0,938	0,115	0,097	17,1
**148**	diarrhea	X	X	✔	✔	X	✔	X	X	X	✔	✔	✔	✔	✔	✔	X	X	0,01	0,004	0,001	4,234	1,7
**149**	diarrhea	X	X	✔	✔	✔	X	✔	✔	X	✔	✔	✔	✔	✔	✔	X	✔	0,013	0,01	0,007	0,133	78,8
**150**	diarrhea	✔	X	✔	✔	✔	✔	✔	✔	X	✔	✔	✔	✔	✔	✔	X	X	0,321	0,144	0,047	0,033	99
**151**	bloody diarrhea	✔	X	✔	✔	X	✔	✔	✔	✔	✔	✔	✔	✔	✔	X	X	X	0,005	0,004	0,004	0,199	62,3
**152**	bloody diarrhea	X	X	✔	✔	✔	X	✔	✔	✔	✔	✔	✔	✔	✔	X	X	X	3,268	0,424	0,574	1,295	32,5
**153**	diarrhea	✔	X	✔	✔	✔	X	✔	✔	✔	✔	✔	✔	✔	✔	X	✔	X	0,011	0,029	0,015	0,238	12,2
**154**	diarrhea	X	X	✔	✔	✔	X	✔	✔	✔	✔	✔	✔	✔	✔	X	X	X	0,002	0,002	0,001	0,28	10
**155**	diarrhea	X	X	✔	✔	X	X	X	X	X	✔	✔	X	✔	✔	X	X	X	0,02	0,021	0,002	2,808	16,5
**156**	diarrhea	X	X	✔	✔	✔	X	✔	✔	X	✔	✔	✔	✔	✔	✔	X	X	0,003	0,013	0,008	0,276	15,8
**157**	diarrhea	✔	X	✔	✔	✔	✔	✔	✔	X	✔	✔	✔	✔	✔	X	✔	X	0,007	0	0,001	0,48	8,8
**158**	diarrhea	X	✔	✔	✔	✔	X	✔	✔	✔	✔	✔	✔	✔	✔	✔	X	X	0,015	0,021	0,006	0,197	29,8
**159**	diarrhea	✔	X	✔	✔	✔	✔	✔	✔	✔	✔	✔	✔	✔	✔	X	X	X	0,345	0,291	0,078	0,164	12,7
**160**	diarrhea	X	X	✔	✔	✔	✔	✔	✔	X	✔	✔	✔	✔	✔	X	X	✔	0,522	0,062	0,008	1,18	16,7
**161**	diarrhea	✔	X	✔	✔	✔	X	✔	✔	X	✔	✔	✔	✔	✔	X	X	✔	1,313	0,459	0,392	0,049	92,3
**162**	diarrhea	X	X	✔	✔	✔	X	✔	✔	X	✔	✔	✔	✔	✔	X	X	✔	0,007	0,002	0,001	0,685	8,1
**163**	diarrhea	X	✔	✔	✔	✔	X	✔	✔	X	✔	✔	✔	✔	✔	✔	X	✔	0,021	0,053	0,023	0,11	37,3
**164**	diarrhea	✔	X	✔	✔	✔	✔	✔	✔	X	✔	✔	✔	✔	✔	X	X	X	0,454	0,321	0,097	6,549	11
**165**	bloody diarrhea	X	X	✔	✔	✔	X	✔	✔	X	✔	✔	✔	✔	✔	X	X	X	0,018	0,012	0,012	2,422	171,9
**166**	diarrhea	✔	X	✔	✔	✔	✔	✔	✔	✔	✔	✔	✔	✔	✔	X	X	X	0,004	0,004	0,002	0,05	16,8
**167**	diarrhea	✔	X	✔	✔	✔	✔	✔	✔	✔	✔	✔	✔	✔	✔	X	X	X	0,031	0,008	0,013	0,055	6, 5
**168**	diarrhea	✔	X	✔	✔	✔	✔	✔	✔	✔	✔	✔	✔	✔	✔	X	X	X	0,011	0,035	0,016	0,08	38,9
**169**	diarrhea	X	✔	✔	✔	✔	✔	✔	✔	X	✔	✔	✔	✔	✔	✔	X	✔	0,018	0,081	0,021	0,198	36,6
**170**	diarrhea	X	X	✔	✔	X	✔	X	X	X	✔	✔	✔	✔	✔	X	X	X	0,015	0,005	0,006	0,282	6,5
**171**	diarrhea	✔	X	✔	✔	✔	✔	✔	✔	✔	✔	✔	✔	✔	✔	X	X	X	0,008	0,004	0,006	0,111	12,4
**172**	diarrhea	X	X	✔	✔	X	✔	X	X	X	✔	✔	✔	✔	✔	X	X	X	12,501	1,184	0,154	0,136	6,4
**173**	diarrhea	✔	X	✔	✔	✔	✔	✔	✔	✔	✔	✔	✔	✔	✔	X	X	X	5,618	5,621	0,429	0,513	112,7
**174**	diarrhea	X	X	✔	✔	X	✔	X	X	X	✔	✔	✔	✔	✔	X	X	X	0,053	0,052	0,051	0,335	7
**175**	diarrhea	X	X	✔	✔	✔	✔	✔	✔	X	✔	✔	✔	✔	✔	X	X	✔	0,002	0,001	0,009	0,525	8,6
**176**	diarrhea	X	X	✔	✔	X	✔	✔	✔	✔	✔	✔	✔	✔	✔	X	X	X	0,008	0,044	0,057	0,07	30,6
**178**	diarrhea	X	X	✔	✔	✔	✔	✔	✔	X	✔	✔	✔	✔	✔	X	X	✔	0,001	0,006	0,002	0,239	6,5
**180**	diarrhea	X	✔	✔	✔	✔	✔	✔	✔	✔	✔	✔	✔	✔	✔	✔	X	X	0,003	0,003	0,004	0,196	14,5
**181**	diarrhea	X	X	✔	✔	X	✔	X	X	X	✔	✔	✔	✔	✔	X	✔	X	0,001	0,019	0,002	0,337	3,3
**182**	bloody diarrhea	X	X	✔	✔	✔	✔	✔	✔	X	✔	✔	✔	✔	✔	X	X	X	0	0	0,001	0,223	10
**183**	bloody diarrhea	✔	X	✔	✔	✔	✔	✔	✔	X	✔	✔	✔	✔	✔	X	X	X	0,021	0,01	0,016	0,441	13,8
**184**	diarrhea	X	X	✔	✔	✔	✔	✔	✔	✔	✔	✔	✔	✔	✔	X	X	X	0,723	0,106	0,029	0,123	26,4
**185**	diarrhea	X	X	✔	✔	✔	✔	✔	✔	✔	✔	✔	✔	✔	✔	X	X	X	0,005	0,005	0,001	0,11	28,5
**186**	unknown	X	X	✔	✔	✔	✔	✔	✔	X	✔	✔	✔	✔	✔	X	X	✔	0,026	0,026	0,005	0,155	59
**187**	diarrhea	✔	X	✔	✔	✔	✔	✔	✔	✔	✔	✔	✔	✔	✔	X	X	X	0,192	0,268	0,075	0,092	43,7
**188**	diarrhea	X	✔	✔	✔	✔	✔	✔	✔	X	✔	✔	✔	✔	✔	✔	X	X	0,005	0,016	0,004	0,147	25,3
**189**	diarrhea	X	✔	✔	✔	✔	✔	✔	✔	✔	✔	✔	✔	✔	✔	✔	X	X	0,002	0,001	0,002	0,116	39,4
**190**	diarrhea	X	X	✔	✔	✔	✔	✔	✔	X	✔	✔	✔	✔	✔	X	X	X	0,006	0,008	0,004	0,029	144,8
**192**	diarrhea	X	✔	✔	✔	✔	✔	✔	✔	✔	✔	✔	✔	✔	✔	✔	X	X	1,124	0,623	0,83	0,051	140,3

**193**	diarrhea	X	X	✔	✔	X	✔	X	X	✔	✔	✔	✔	X	✔	X	X	X	0,04	0,017	0,184	0,179	30,6
**194**	diarrhea	X	X	✔	✔	✔	X	✔	✔	✔	✔	✔	✔	✔	✔	X	X	X	0,003	0,011	0	0,269	25,8
**195**	diarrhea	X	✔	✔	✔	✔	✔	✔	✔	✔	✔	✔	✔	✔	✔	✔	X	X	0,132	0,003	0,01	0,261	23,8
**196**	diarrhea	X	X	✔	✔	✔	X	✔	✔	X	✔	✔	✔	✔	✔	X	X		0,01	0,007	0,009	0,494	111,4
**197**	diarrhea	X	X	✔	✔	✔	X	✔	✔	✔	✔	✔	✔	✔	✔	X	X	X	0,038	0,014	0,012	2,13	30,6
**198**	diarrhea	X	X	✔	✔	✔	X	✔	✔	✔	✔	✔	✔	✔	✔	X	X	X	0,003	0,013	0,001	1,53	37,5
**201**	diarrhea	X	X	✔	✔	✔	X	✔	✔	X	✔	✔	✔	✔	✔	X	X		0,006	0,006	0,011	0,536	10,6
**203**	diarrhea	✔	X	✔	✔	✔	✔	✔	✔	✔	✔	✔	✔	✔	✔	X	X	X	0,008	0,016	0,031	0,81	8,7
**13**	diarrhea	✔	X	✔	✔	✔	✔	✔	✔	✔	✔	✔	✔	✔	✔	X	X	X	0	0	0	0	0
**81-176**	diarrhea	X	X	✔	✔	✔	✔	✔	✔	✔	✔	✔	✔	✔	✔	X	✔	X	0,015	0,003	0	2,056	14,4
**81116**	diarrhea	X	✔	✔	✔	✔	✔	✔	✔	✔	✔	✔	✔	✔	✔	✔	X	X	0,005	0,018	0,003	1,098	22

Supplementary Figure 1. Distribution of rates of adhesion and invasion (Table S2) in all the strains (A) and only in the 23 strains isolated from bloody diarrhoea (B).

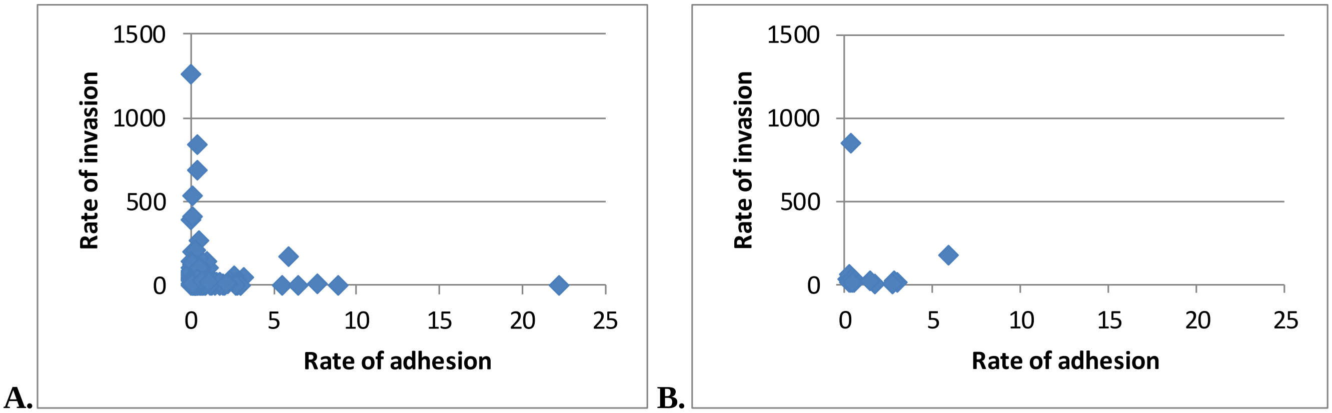

A. **B.**

Supplementary Figure 2. The expression changes of the flagellar regulatory cascades of the highly invasive *C. jejuni* strain 2006-119 during the invasion process (Gilbreath et al. 2011)

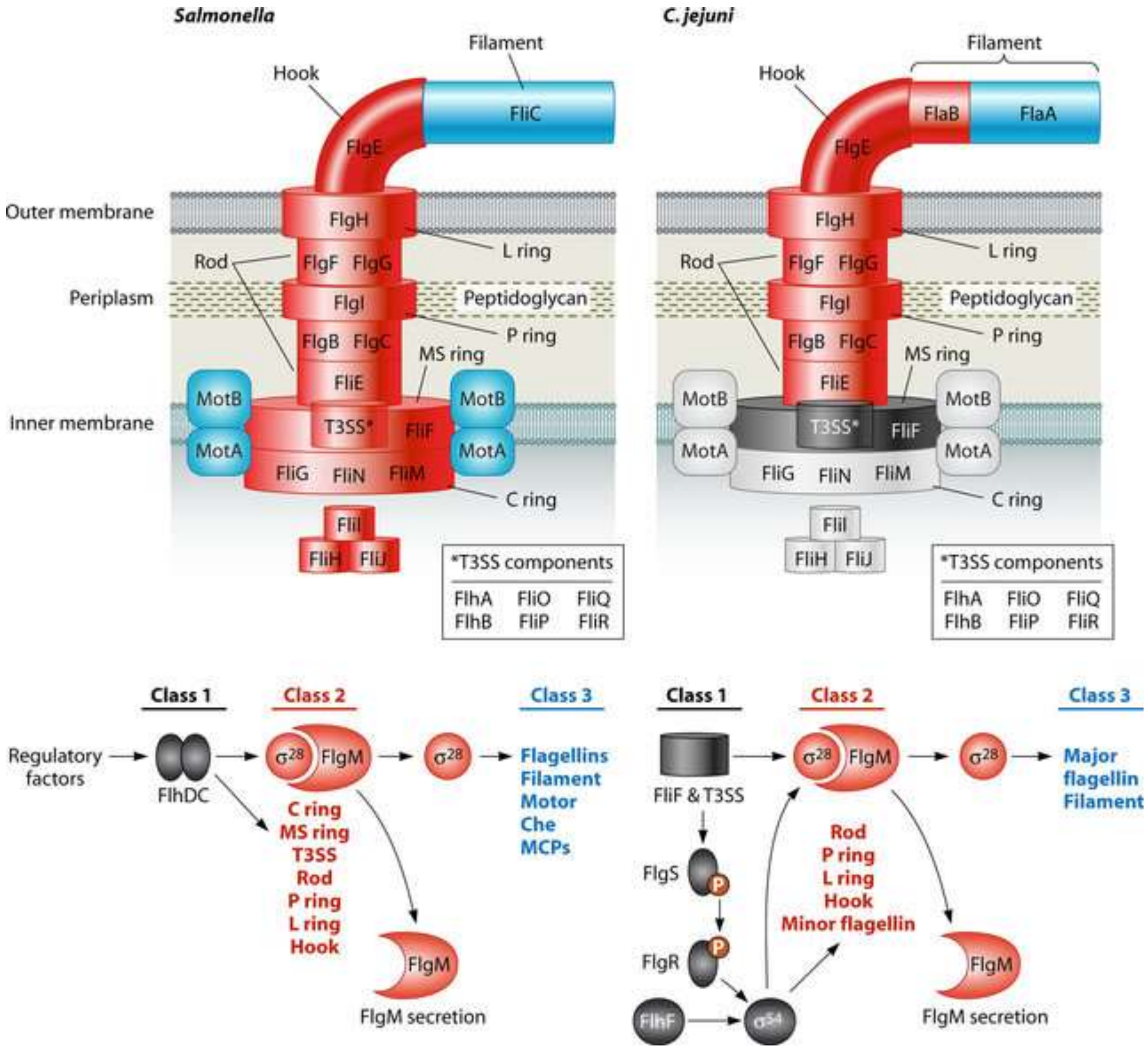

Supplement: Supplementary file 1 [file microorganisms-08-00531-s001.pdf]
